# *Cornus officinalis* Ethanolic Extract with Potential Anti-Allergic, Anti-Inflammatory, and Antioxidant Activities

**DOI:** 10.3390/nu12113317

**Published:** 2020-10-29

**Authors:** Yixian Quah, Seung-Jin Lee, Eon-Bee Lee, Biruk Tesfaye Birhanu, Md. Sekendar Ali, Muhammad Aleem Abbas, Naila Boby, Zi-Eum Im, Seung-Chun Park

**Affiliations:** 1 Laboratory of Veterinary Pharmacokinetics and Pharmacodynamics, College of Veterinary Medicine, Kyungpook National University, Daegu 41566, Korea; im.yixianquah@gmail.com (Y.Q.); dvmleesj@naver.com (S.-J.L.); eonbee@gmail.com (E.-B.L.); btbtes@gmail.com (B.T.B.); alipharm2000@gmail.com (M.S.A.); syedaleemabbas77@gmail.com (M.A.A.); nailaboby1584@gmail.com (N.B.); 2 Development and Reproductive Toxicology Research Group, Korea Institute of Toxicology, Daejeon 34114, Korea; 3 Department of Biomedical Science and Department of Pharmacology, School of Medicine, Brain Science and Engineering Institute, Kyungpook National University, Daegu 41944, Korea; 4 Department of Pharmacy, International Islamic University Chittagong, Kumira, Chittagong 4318, Bangladesh; 5 Forest Resources Development Institute of Gyeongsangbuk-do, Andong-si, Gyeongsangbuk-do 36605, Korea; zium78@korea.kr

**Keywords:** anti-inflammatory activity, antioxidant activity, atopic dermatitis, *Cornus officinalis*, molecular docking, human high-affinity IgE receptors

## Abstract

Atopic dermatitis (AD) is an allergic and chronic inflammatory skin disease. The present study investigates the anti-allergic, antioxidant, and anti-inflammatory activities of the ethanolic extract of *Cornus officinalis* (COFE) for possible applications in the treatment of AD. COFE inhibits the release of β-hexosaminidase from RBL-2H3 cells sensitized with the dinitrophenyl-immunoglobulin E (IgE-DNP) antibody after stimulation with dinitrophenyl-human serum albumin (DNP-HSA) in a concentration-dependent manner (IC_50_ = 0.178 mg/mL). Antioxidant activity determined using 2,2-diphenyl-1-picrylhydrazyl (DPPH) radical scavenging activity, ferric reducing antioxidant power assay, and 2,2′-azino-bis(3-ethylbenzothiazoline-6-sulfonic acid) (ABTS) scavenging activity, result in EC_50_ values of 1.82, 10.76, and 0.6 mg/mL, respectively. Moreover, the extract significantly inhibits lipopolysaccharide (LPS)-induced nitric oxide (NO) production and the mRNA expression of iNOS and pro-inflammatory cytokines (IL-1β, IL-6, and TNF-α) through attenuation of NF-κB activation in RAW 264.7 cells. COFE significantly inhibits TNF-α-induced apoptosis in HaCaT cells without cytotoxic effects (*p* < 0.05). Furthermore, 2-furancarboxaldehyde and loganin are identified by gas chromatography/mass spectrometry (GC-MS) and liquid chromatography with tandem mass spectrometry (LC-MS/MS) analysis, respectively, as the major compounds. Molecular docking analysis shows that loganin, cornuside, and naringenin 7-O-β-D-glucoside could potentially disrupt the binding of IgE to human high-affinity IgE receptors (FceRI). Our results suggest that COFE might possess potential inhibitory effects on allergic responses, oxidative stress, and inflammatory responses.

## 1. Introduction

Atopic dermatitis (AD) is an allergic and chronic skin inflammation condition characterized by pruritic eczema and mechanical skin injury caused by scratching, elevated serum IgE levels, and markedly increased immune cells levels (eosinophils, mast cells, and lymphocytes) [1]. Particularly, T helper 2 (Th2) cells are involved in AD development [2]. Th2 cells release cytokines such as interleukin (IL)-4, IL-5, IL-10, and IL-13, resulting in the activation and proliferation of eosinophils and mast cells. The resultant itching, skin scratching, mechanical skin barrier injury, and activation of immune cells, lead to further Th2 cell activation and itching [3].

*Cornus officinalis* is a dogwood species native to Eastern Asia (Korea, China, and Japan). Its fruit has been used in traditional medicine for backache, hypertension, and polyuria in Korea [4]. The protective effect of *C. officinalis*, used in herbal mixture with four other plant extracts, against atopic dermatitis in BALB/C mice was recently reported [5]. The major component of the reported herbal mixture was *C. officinalis* extract, constituting 50% of the formulation. Besides, a recent review on this medicinal plant discussed its biological properties such as antioxidant, anti-inflammatory, renal and hepatic protective, antimicrobial, and immunomodulatory effects [6] which all play a substantial role in modulating pathological mechanism in AD.

Previous studies almost exclusively focused on the anti-atopic, antioxidant, and anti-inflammatory effects of the herbal mixture but not on *C. officinalis* used individually in the in vitro studies for AD. This study is the first to report the potency and efficacy of the anti-atopic effects of the *C. officinalis* extract in an in vitro model of AD.

## 2. Materials and Methods

### 2.1. Sample Preparation and Extraction

To prepare the ethanolic extract of *C. officinalis*, dried fruits of *C. officinalis* (50 g) (Sansooyoo, Korea) were extracted three times with 70% ethanol (500 mL) at 65 °C for 4 h using a heating mantle. The filtered supernatant was concentrated using a rotary evaporator. The dense supernatant was solidified with a vacuum freeze dryer. The yield of the ethanolic extraction of *C. officinalis* (COFE) was approximately 14.33% of the dried material. COFE was dissolved in dimethyl sulfoxide (DMSO, Sigma-Aldrich, St. Louis, MO, USA) and stored as a stock solution at −20 °C until use.

### 2.2. β-Hexosaminidase Release

The inhibition of β-hexosaminidase release from a rat basophilic leukemia cell line (RBL-2H3) was evaluated using the method described by Juckmeta et al. [7] with some modifications. Briefly, RBL-2H3 cells were seeded at 5 × 10^5^ cells/mL and incubated for 1.5 h. RBL-2H3 cells were sensitized with anti-dinitrophenyl-immunoglobulin E (anti-DNP IgE; 0.45 μg/mL) and incubated for 24 h. The cells were washed with Siraganian buffer. Siraganian buffer (160 μL) was added and the cells were incubated for 10 min at 37 °C. Different concentrations of COFE (20 μL) were added to each well to reach a final concentration of 0, 0.003, 0.01, 0.03, 0.1, and 0.3 mg/mL, then the cells were incubated for 10 min. Next, 20 μL of dinitrophenyl-human serum albumin (DNP-HSA, final concentration 10 μg/mL) was added to each well and the cells were incubated for another 20 min to stimulate cell degranulation. The supernatants were transferred to 96-well plates at 50 μL/well and incubated with 50 μL of *p*-nitrophenyl-*N*-acetyl-b-D-glucosaminide (PNAG, 1 mM) in 0.1 M citrate buffer (pH 4.5) at 37 °C for 3 h. The reaction was stopped by the addition of 200 μL of the stop solution. The absorbance was measured at 405 nm. The percentage of β-hexosaminidase release was calculated as a percentage of the total β-hexosaminidase content. A brief experimental timeline of this assay is illustrated in Supplementary Figure S1.

### 2.3. Antioxidant Activity

#### 2.3.1. 2,2-Diphenyl-1-picrylhydrazyl (DPPH) Free Radical Scavenging Activity

The extracts scavenging activity against DPPH free radicals was evaluated using the method described by Chang et al. [8]; ascorbic acid was used as a positive control. The presence of DPPH free radicals was measured at 517 nm.

#### 2.3.2. Ferric Reducing Antioxidant Power (FRAP) Assay

The ferric reducing ability of tissues was determined using a modified FRAP method [9]. The absorbance of the reaction mixture was read at 595 nm. The results were expressed as μM Trolox equivalent (TE), as E_max_ value, which is the value at which the maximal effect of the sample was obtained, and as EC_50_ value, which is the TE of the sample required to cause a 50% reduction in the FRAP value.

#### 2.3.3. Trolox Equivalent Antioxidant Capacity (TEAC) Assay

The TEAC levels were determined with the method of Rufino et al. [10], with modifications. Trolox (10–1000 μmol TE) was used to generate the standard curve. Ten microliters of each sample were added to 90 μL of an ABTS^•^ solution and the absorbance was measured at 734 nm. The results were expressed in μmol TE, as E_max_ value, and EC_50_ value, which is the TE of the sample required for the inhibition of 50% ABTS free radical activity.

### 2.4. Nitric Oxide (NO) Assay

RAW 264.7 cells (Korean Cell Line Bank, Seoul, Korea) at 4 × 10^5^ cells/well were allowed to adhere for 24 h until 80% confluency was reached. After 30 min of treatment of different extract concentrations, the cells were incubated with lipopolysaccharide (LPS) treatment (1 µg/mL) for 18 h. NO production in the cell culture medium was measured as nitrite (NO_2_) at 540 nm and quantified from a standard curve generated from sodium nitrite (NaNO_2_). A brief experimental timeline of this assay was illustrated in Supplementary Figure S1. The cytotoxicity of the extracts in RAW 264.7 cells was determined using an 3-(4,5-dimethylthiazol-2-yl)-2,5-diphenyl tetrazolium bromide (MTT) assay [11].

### 2.5. Total RNA Extraction and Quantitative RT-PCR

Total RNA was extracted from cells using TRIzol reagent and used for cDNA synthesis with the cDNA EcoDry Premix (Takara, Shiga, Japan). Relative mRNA levels were determined by cycler CFX96 (Bio-Rad). PCR amplification was performed in triplicate with the following specific primers: IL-1β forward (F): TGAGCACCTTCTTTTCCTTCA and reverse (R): TTGTCTAATGGGAACGTCACAC; IL-6 F: TAATTCATATCTTCAACCAAGAGG and R: TGGTCCTTAGCCACTCCTTC; TNF-α F: CTGTAGCCCACGTCGTAGC and R: GGTTGTCTTTGAGATCCATGC; iNOS F: TGTGGCTACCACATTGAAGAA and R: TCATGATAACGTTTCTGGCTCTT; and β-actin F: GTCATCACTATTGGCAACGAG and R: TTGGCATAGAGGTCTTTACGG. Real-time PCR (RT-PCR) was performed using the following cycling conditions: enzyme activation and initial denaturation for 5 min at 95 °C and 40 cycles of amplification at 95 °C for 10 s followed by 55 °C (IL-1 β and IL-6), 58 °C (iNOS), or 62 °C (TNF-α) for 20 s.

### 2.6. Western Blot Analysis

RAW 264.7 cells were treated for 60 min with COFE (0–0.3 mg/mL) and then treated with LPS (1 μg/mL) for 30 min (for IkBα and p-p65 protein determination), or treated with LPS (1 μg/mL) for 18 h (for iNOS protein determination) (Supplementary Figure S1). At the end of the LPS treatment, Western blot analysis was conducted according to a reported method [12], with slight modification. Briefly, after washing cells with ice-cold PBS, total proteins were extracted from the cell pellets using the PRO-PREP protein extraction solution (iNtRON Biotechnology, Seongnam, Korea) according to the manufacturer’s instruction. The lysates were centrifuged at 16,000× *g* for 10 min at 4 °C and stored at −20 °C until use. Protein was then measured using the Pierce™ BCA assay kit (ThermoFisher Scientific, Waltham, United States), with bovine serum albumin as a standard and then separated by sodium dodecyl sulfate polyacrylamide gel electrophoresis (SDS-PAGE) in 10% gels, and transferred to polyvinylidene fluoride (PVDF) membranes. Membranes were blocked with 5% bovine serum albumin (BSA) in tris-buffered saline with tween-20 (TBST) for 1 h at room temperature followed by incubating with appropriate primary antibodies for 90 min at room temperature. Membranes were washed three times and incubated for an additional 60 min with horseradish peroxidase-conjugated secondary antibody. The expression of IkBα and p-p65 (1:1000) (both were purchased from Cell Signaling Technology, Danvers, MA, USA) and β-actin (Santa Cruz Biotechnology, Dallas, TX, USA) as a loading control were visualized using Thermo Scientific™ Pierce™ ECL Western Blotting Substrate (ThermoFisher Scientific, Waltham, MA, USA).

### 2.7. Flow Cytometric Analysis

The MTT assay was used to examine the cytotoxicity of COFE in HaCaT cells [11]. The absorbance was measured at 570 nm. After TNF-α (20 ng/mL) treatment for 1 h, the cells were incubated with different extract concentrations for 18 h. The activation of caspases 3 and 7 in HaCaT cells was detected using the Muse Caspase-3/7 assay kit with a Muse Cell Analyzer (EMD Millipore, Billerica, MA, USA) according to the manufacturer’s protocol. The data from the Muse Cell Analyzer were analyzed using Muse 1.4 software (Luminex Corporation, Austin, TX, USA). A brief experimental timeline of this assay was illustrated in Supplementary Figure S1.

### 2.8. Gas Chromatography/Mass Spectrometry (GC-MS) and Liquid Chromatography with Tandem Mass Spectrometry (LC-MS/MS) Analysis

The GC-MS analysis of COFE was performed by using an HP 6890 Plus GC gas chromatograph with a mass selective detector (MSD; HP 5973, Hewlett-Packard, California, United States). The samples were diluted 1:1000 (*v*:*v*) with HPLC-grade dichloromethane. The samples (1 μL) were injected into an HP-5 column. The GC oven temperature was set at 50 °C for 4 min, increased to 280 °C at a rate of 4 °C/min, and held at the final temperature for 2 min. The velocity of the carrier gas, 99.99% He, was 0.7 mL/min. Quantitative analysis was performed by using the area normalization method.

LC-MS analysis was performed on a Thermo Accela UHPLC system (Thermo Fisher Scientific, San Jose, CA, USA). The samples were separated on a Waters BEH C18 column (2.1 × 150 mm, 1.7 μm) at room temperature. The mobile phase consisted of water (A) and acetonitrile (B), both with 0.1% formic acid added. The elution gradient was set as follows: 5% B (0 min), 5% B (1 min), 70% B (20 min), 100% B (24 min), and 100% B (27 min). The flow rate was 400 μL/min and the sample loading volume was 1 μL. The UHPLC was coupled to an LTQ-Orbitrap XL hybrid mass spectrometer (Thermo Electron, Bremen, Germany) via an ESI interface. The samples were analyzed in positive ion mode and the conditions of the ESI source were the same as previously used [13].

### 2.9. In Vivo Toxicity Evaluation

Ten female 8-week-old Sprague-Dawley rats were obtained from Orient Bio Inc. (Gyeonggi-do, Korea). The acute toxicity study was performed by using a previously reported method [14] with slight modifications. Five rats per group were randomly assigned to the control and test groups. A single dose of COFE (2000 mg/kg of body weight) was administered intragastrically according to the OECD test guideline 423 [15]. A standard pellet diet (Hyochang Science, Daegu, Korea) and distilled water were provided ad libitum. The animals were under constant observation for abnormal signs and symptoms for the first 12 h after COFE administration and subsequently observed once per day for 2 weeks. The changes in body weight and food and water intake were measured twice a week for 14 days after treatment. The animals were sacrificed after the experimental period and the major organs were collected and inspected for gross lesions.

### 2.10. Molecular Docking

To investigate the binding mode of the loganin, cornuside, and naringenin 7-O-β-D-glucoside to human high-affinity IgE receptors (FceRI), a molecular docking analysis was performed using the FceRI-IgE complex structure data with the protein data bank (PDB) [16] ID 2Y7Q [17]. The conformations of loganin (ZINC000003978792) and naringenin 7-O-β-D-glucoside (ZINC4097895) were generated using a conformational search against the ZINC docking database, University of California, San Francisco (UCSF). The cornuside (InChI Key: SMTKSCGLXONVGL-UHFFFAOYSA-N) conformation was searched against the PubChem database. The ligand file in Structure Data File (SDF) format was converted to MOL format using the Openbabel software. The co-crystallized structures were prepared using UCSF Chimera (Chimera, Version 1.12, RBVI, San Francisco, CA, USA) and iGEMDOCK (Version 2.1; NCTU, Hsinchu City, Taiwan). Molecular docking was performed using iGEMDOCK with the accurate docking mode. The best-docked poses were further analyzed, and 3D structure images were prepared using UCSF Chimera.

### 2.11. Statistical Analysis

The IC_50_ and EC_50_ values were calculated using Prism (GraphPad Software Inc., La Jolla, CA, USA). Statistical differences were assessed by one-way analysis of variance and Duncan’s multiple comparisons test using Statistical Analysis Systems software (SAS Institute, Cary, NC, USA). Values of *p* < 0.05 were considered statistically significant.

## 3. Results

### 3.1. Effect of COFE on DNP/IgE-Induced Degranulation in RBL-2H3 Cells

**Figure 1:**
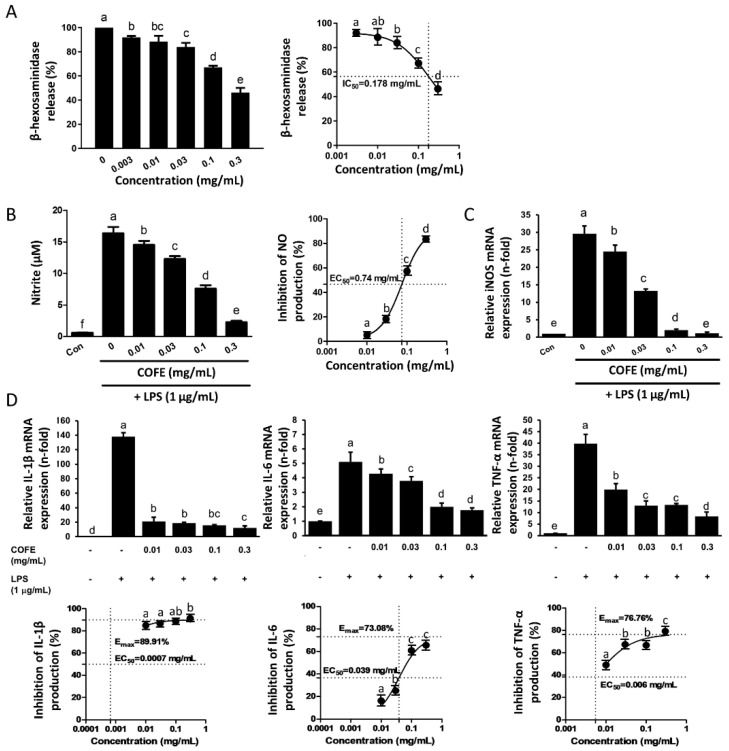
Anti-allergic and anti-inflammatory activity of ethanolic extract of *Cornus officinalis* (COFE). (**A**) Evaluation of the β-hexosaminidase release from RBL-2H3 cells treated with COFE (left) and the IC_50_ of COFE for β-hexosaminidase release (right). (**B**) Effect of COFE on lipopolysaccharide (LPS)-induced nitric oxide (NO) production. (**C**) iNOS mRNA expression. (**D**) Anti-inflammatory cytokine gene expression. Values are expressed as the mean ± SD of three independent experiments. Letters (a–e) indicate significantly different values (*p* < 0.05), as determined by Duncan’s multiple comparison test.

COFE suppressed the release of β-hexosaminidase from RBL-2H3 cells in a concentration-dependent manner (Figure 1A). A dose of 0.3 mg/mL of COFE significantly inhibited β-hexosaminidase release (46%) (*p* < 0.05). The IC_50_ value of COFE for β-hexosaminidase release inhibition was 0.178 mg/mL, showing that the potential anti-atopic activity occurred through the suppression of the IgE-induced degranulation of RBL-2H3 cells.

### 3.2. Antioxidant Activity of COFE

Supplementary Figure S2 shows the DPPH radical scavenging activity of COFE. COFE showed concentration-dependent DPPH radical scavenging activity from 0.1 to 10 mg/mL. Its EC_50_ value was 1.82 mg/mL, which is approximately six times higher than that of ascorbic acid (0.30 mg/mL). The E_max_ values of COFE were 3.15 mM TE and 3.23 mM TE in the FRAP assay and ABTS assay, respectively. Its EC_50_ values were 10.76 mg/mL and 0.60 mg/mL in the FRAP assay and ABTS assay, respectively.

### 3.3. Effect of COFE on LPS-Induced NO Production and Pro-Inflammatory Cytokines in RAW 264.7 Cells

COFE significantly inhibited LPS-induced NO production in RAW 264.7 cells in a concentration-dependent manner (0.01–0.3 mg/mL) (*p* < 0.05) with an EC_50_ value of 0.74 mg/mL (Figure 1B). Similarly, COFE attenuated LPS-induced iNOS mRNA expression in RAW 264.7 cells in a concentration-dependent manner, indicating a positive correlation between the inhibition of NO production and the suppression of iNOS mRNA expression (Figure 1C). At the tested concentrations, COFE did not exert any cytotoxic effects on RAW 264.7 cells (data not shown). Furthermore, COFE inhibited LPS-induced IL-1β, IL-6, and TNF-α mRNA expression in RAW 264.7 cells in a concentration-dependent manner (0.01–0.3 mg/mL), with E_max_ values ranging from 73% to 90% (Figure 1D). Furthermore, the EC_50_ values for the inhibition of IL-1β, IL-6, and TNF-α were 0.0007, 0.039, and 0.006 mg/mL, respectively.

### 3.4. Effect of COFE on NF-κB Stimulation

**Figure 2:**
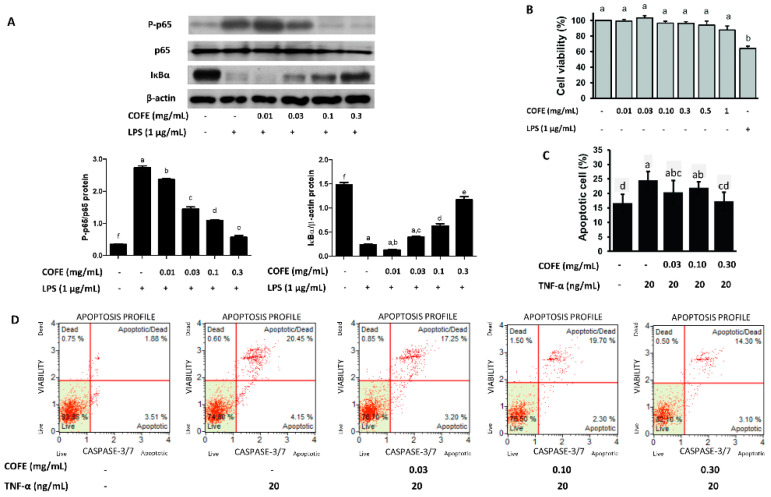
Effect of COFE on LPS-induced NF-κB activation in RAW 264.7 cells (**A**), cell viability (**B**), and TNF-α-induced apoptotic death in HaCaT cells (**C**,**D**). (**A**) Cells were treated with COFE (0–0.3 mg/mL) for 60 min and then treated with LPS (1 µg/mL) for 30 min. The levels of total IκBα and phospho-p65 (P-p65) were determined by Western blotting of the total protein of cell lysates (*n* = 3). β-actin was used as a loading control. (**B**) The viability of HaCaT cells treated with increasing concentrations of COFE (0–1 mg/mL) for 24 h was evaluated using an MTT assay; LPS was used as a control. Each sample was assayed in triplicate at each concentration. (**C**) After TNF-α (20 ng/mL) treatment for 1 h, cells were incubated with the indicated concentrations of extract for 18 h. TNF-α-induced apoptosis was detected by flow cytometric analysis. The data show healthy cells (annexin-V negative and caspase 3/7 and 7-ADD negative (lower left)), early apoptotic cells (positive for annexin-V and caspase 3/7 and negative for 7-ADD (lower right)), late apoptotic/dead cells (both annexin V and caspase 3/7 and 7-ADD positive (upper right)), and necrotic cells (only 7-ADD positive (upper left)) (*n* = 3). (**D**) Total apoptotic cells (early apoptotic and late apoptotic cells) were quantified by positive staining for annexin-V or caspase 3/7 activity. Values are expressed as the mean ± SD of three independent experiments. Letters (a–f) indicate significantly different values (*p* < 0.05), as determined by Duncan’s multiple comparison test.

Western blot analysis showed that LPS reduced the cytosolic IκBα expression but COFE increased it in a concentration-dependent manner (Figure 2A). Comparably, COFE inhibited LPS-induced cytosolic p-p65 expression. The expression of iNOS protein also showed significant reduction compared to the LPS induced group (Supplementary Figure S3). Thus, the anti-inflammatory activity of COFE occurs through the inhibition of NF-κB activation.

### 3.5. Effect of COFE on TNF-α Induced Apoptosis in HaCaT Cells

To assess the suitability of COFE for the treatment of human AD, cytotoxicity and apoptosis were examined in human keratinocytes. Treating HaCaT cells with COFE (0–1 mg/mL) for 24 h did not affect their viability (Figure 2B). The impact of COFE on TNF-α-induced apoptosis in HaCaT cells was evaluated through cytofluorometric analysis. TNF-α-induced apoptosis was estimated through the net change in the percentage of total apoptotic cells compared with the controls (Figure 2C). After caspase 3/7 antibody treatment, TNF-α-treated samples exhibited approximately 1.5× apoptotic/dead and apoptotic cells in comparison with untreated cells (Figure 2D). COFE significantly reduced TNF-α-induced cell apoptosis (*p* < 0.05).

### 3.6. Identification of the Constituent Compounds of COFE

**Figure 3:**
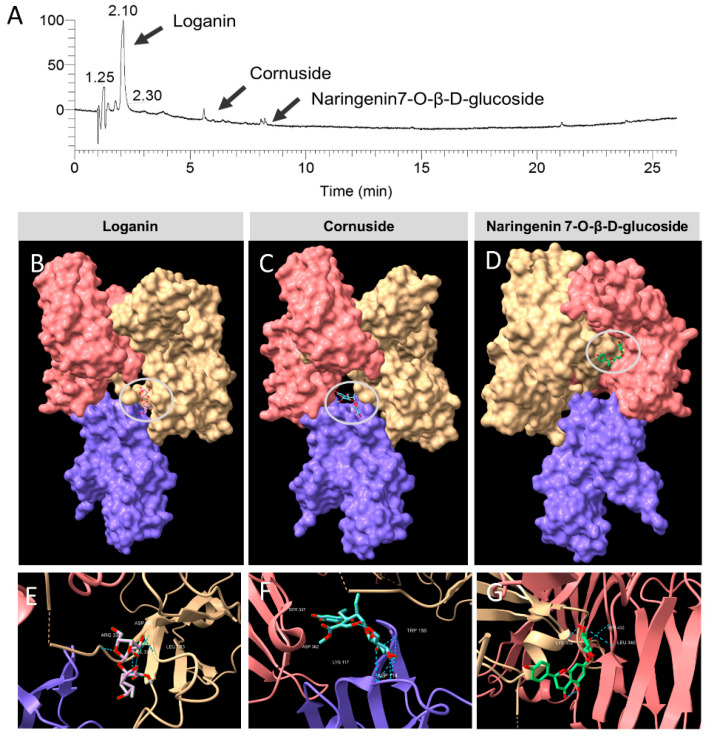
Liquid chromatography with tandem mass spectrometry (LC-MS/MS) COFE (**A**) and molecular docking analysis of the FceRI-IgE complex (**B**–**G**). The best-docked position showing the interaction sites of loganin (**B**), cornuside (**C**), and naringenin 7-O-β-D-glucoside (**D**) in the FceRI-IgE complex. The blue dotted lines represent the hydrogen bonds between the amino acid residues of the FceRI-IgE complex and loganin (ARG334, VAL336, ASP362, LEU363, ALA364), cornuside (SER337, ASP362, LYS117, ASP114, TRP156), and naringenin 7-O-β-D-glucoside (LYS302, ARG342, LEU340, ARG431, ALA338) (**E**–**G**).

Table 1 shows the main constituents of COFE identified by GC-MS analysis, namely 4,6-cycloheptatrien-1-one, malic acid, 2-furancarbox-aldehyde, and 2,3-dihydro-3,5-dihydroxy-6-methyl-4H-pyran-4-one. The compounds responsible for the physiological effects in COFE were analyzed by LC-MS/MS and identified as loganin, cornuside, and naringenin 7-O-β-D-glucoside (Figure 3A). Loganin, a natural flavonoid, was the major component of COFE.

### 3.7. In Vivo Toxicity Evaluation of COFE

Animals displayed no clinical signs or mortality up to 14 days after the administration. The same procedure was carried out on three female rats and a similar observation was obtained. Therefore, the acute toxic class method, following the flow chart of LD_50_ cut-off, confirmed COFE as a category 5 substance in the Globally Harmonized System of Classification and Labeling of Chemicals (GHS).

### 3.8. Molecular Docking

By integrating the data obtained from LC-MS analysis with the in silico molecular docking analysis, the major bioactive components (loganin, cornuside, and naringenin 7-O-β-D-glucoside) found in COFE were found to interact with the FceRI-IgE complex. Figure 3B–D shows the loganin, cornuside, and naringenin 7-O-β-D-glucoside binding to the 3D structure of the FceRI-IgE complex. Figure 3E–G shows the amino acid residues associated with the binding of the compounds through hydrogen bonding. The binding energies of the FceRI-IgE complex with the compounds according to molecular docking analysis are shown in Table 2.

## 4. Discussion

The anti-allergic potential of COFE was assessed with RBL-2H3 cells (Figure 1), which are commonly used to study allergy and inflammation reactions because they express high-affinity IgE receptors (FceRI) [5]. β-Hexosaminidase was used as an indicator of degranulation in RBL-2H3 cells sensitized by exposure to IgE-DNP antibody after stimulation with DNP-HSA [18]. Our results show COFE inhibits β-Hexosaminidase release with an IC_50_ of 0.178 mg/mL which is about 60- and 1.4-fold lower than that of ethanol extracts from mulberry fruit [19] and *Arctium lappa* fruit [20], respectively. Antioxidants potentially exert beneficial effects in the treatment of AD by inhibiting the oxidative stress which is involved in the pathophysiology of the acute exacerbation of AD [21]. However, the antioxidant activity of COFE was lower than that reported by Hwang and co-workers [4]. This may be due to the concentration of bioactive compounds in the plant, which could be influenced by photoperiodic, temperature, and geographical differences [22].

**Figure 4:**
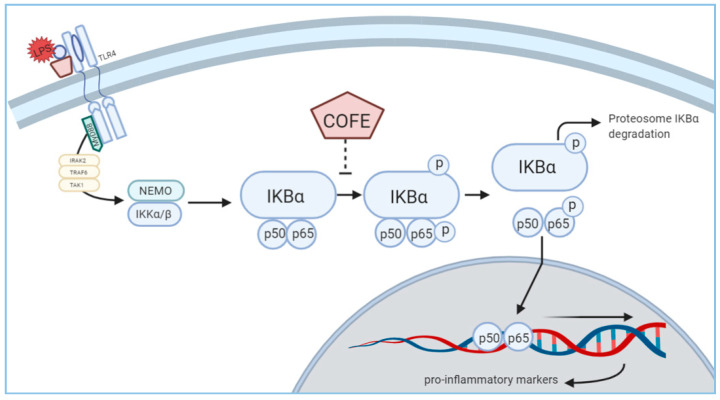
Illustration of the mechanism of action of COFE through NF-κB suppression in RAW 264.7 cells treated with LPS. TLR4: Toll-like receptor 4; IRAK2: Interleukin 1 Receptor Associated Kinase 2; TRAF6: Tumor necrosis factor receptor (TNFR)-associated factor 6; TAK1: Transforming growth factor β-activated kinase 1; MyD88: Myeloid differentiation primary response 88; NEMO: nuclear factor kappa-B essential modulator; IKKα/β: inhibitor of nuclear factor kappa-B kinase subunit α/β; IKBα: nuclear factor of kappa light polypeptide gene enhancer in B-cells inhibitor alpha; p65/p50: nuclear factor NF-kappa-B p65/p50 subunit.

Macrophages play crucial roles in acute inflammation and persistence of proinflammatory cytokines, which lead to chronic inflammation conditions including AD [23]. We found that COFE significantly inhibits LPS-induced NO production, iNOS mRNA expression (Figure 1B,C), and iNOS protein expression in RAW 264.7 cells (Figure S3). Besides, COFE consistently suppresses the expression of IL-1β, IL-6, and TNF-α, suggesting that the upstream signaling molecules (such as NF-κB) could be the determining step in the anti-inflammatory response induced by COFE (Figure 4). Further studies are needed to confirm the use of COFE as a therapeutic agent for AD and various inflammatory diseases through the reduction of pro-inflammatory cytokines. Several plant extracts with potential anti-inflammatory activity may be suitable for the treatment of immune disorders like AD [24].

COFE significantly inhibited TNF-α-induced apoptosis in HaCaT cells. TNF-α and pro-inflammatory cytokines are secreted by macrophages and mast cells and promote apoptosis in HaCaT cells by binding to the TNF-receptor 1 [25]. Considering the deterioration of skin lesions by inflammatory mediators that are released from various skin cells during the inflammatory process, COFE could be useful for the management of AD without acute oral toxicity.

Oxidative stress is associated with chronic skin inflammation and plays a role in the pathogenesis of AD [26]. Reactive oxygen species (ROS) can modify DNA, lipids, and proteins [26]. NO production is one of the biomarkers of lipid peroxidation resulting from oxidative stress [27]. A previous report stated that the increase in oxidative stress in AD was due to an increase in lipid peroxidation and a decrease in antioxidant levels [21]. The antioxidant properties of COFE (Figure S2) could reduce oxidative stress and thus disrupt the pro-inflammatory microenvironment through ROS-mediated signaling events.

Loganin, the main bioactive constituent in COFE, synergistically contributes to the inhibition of oxidative stress, inflammation, and metabolic disorders [28]. It may directly promote the differentiation of MC3T3-E1 cells and inhibit their apoptosis [29]. Cornuside attenuates LPS-induced inflammatory cytokines through the inhibition of NF-κB activation in RAW 264.7 cells [30]. Moreover, cornuside shows remarkable antioxidant activity and inhibits isoproterenol-induced myocardial cell necrosis [31]. Interestingly, naringenin 7-*O*-β-D-glucoside (identified and isolated from *Marrubium globosum* Montbr. et Auch. ex Benth. ssp. *libanoticum* Boiss. (Lamiaceae), a medicinal plant used to treat inflammatory diseases and asthma), shows antibacterial activity in several bacterial strains [32]. Besides, naringenin 7-*O*-β-D-glucoside has been reported for its antioxidant activity [33]. These compounds may act synergistically to confer various bioactivities to COFE. Molecular docking analysis was carried out to elucidate the interaction between the FceR1-IgE complex and the bioactive compounds identified in COFE.

The best poses of FceR1-IgE docked with loganin, cornuside, and naringenin 7-*O*-β-D-glucoside were determined. Interestingly, the docking analysis showed that loganin, being the major component found in this extract, binds to IgE through hydrogen bonds with ARG334, VAL336, ASP362, LEU363, and ALA364 (Figure 3E). It is noteworthy that, in humans, the disruption of the glycosylation site at asparagine-394 (ASN394) in the Cε3 domain nullifies the binding of IgE to FceR1 [34]. From our simulation analysis, the binding site of loganin to the complex was the nearest to ASN394, which might indirectly disrupt the formation of the FceR1-IgE complex. Conversely, cornuside possessed the lowest binding energy (−141.1 Kcal) among the compounds tested (Table 2). A lower energy value represents a higher receptor-ligand binding affinity. In other words, cornuside forms a more stable receptor-ligand complex with FceR1-IgE compared to loganin and naringenin 7-*O*-β-D-glucoside. The region where naringenin 7-*O*-β-D-glucoside interacts with IgE was further analyzed by the protein sequences in ChimeraX. The protein sequences were then searched against the conserved domain database (CDD) [35]. We found that naringenin 7-*O*-β-D-glucoside binds to the CH2 domain (second constant Ig domain of the heavy chain) of IgE, cd05847. Therefore, the binding of naringenin 7-*O*-β-D-glucoside could prevent the conformation change of IgE, subsequently disrupt the engagement of the second Cε3 domain to FceR1, and lead to a reduction of skin inflammation.

Our findings suggest diverse potential dermatologic and cosmetic applications for COFE. In silico findings suggest that the bioactive compounds found in COFE impede skin inflammation by disrupting the binding of IgE to the human high-affinity IgE receptors (FceRI). Since our study relies only on in vitro assays, further studies using animal models or artificial skin models would confirm the therapeutic effects and mechanisms of action of COFE.

## 5. Conclusions

Our results show that COFE might exert inhibitory effects on oxidative stress, allergic responses, and inflammatory responses. Additionally, loganin, cornuside, and naringenin 7-*O*-β-D-glucoside were identified as compounds potentially responsible for these effects. COFE showed antioxidant, anti-allergic, and anti-inflammatory effects that could potentially be leveraged to treat AD, yet did not exert cytotoxicity or acute oral toxicity. Our findings suggest that COFE could be used in the development of preventative and treatment therapies for AD.

## Figures and Tables

**Table 1:** Gas chromatography/mass spectrometry (GC-MS) analysis of compounds of the ethanolic extract of *C. officinalis*.

RT (Retention Time)	Formula	%	ID	Activity
34.21	C_6_H_8_O_4_	4.58	2,3-dihydro-3,5-dihydroxy-6-methyl-4H-pyran-4-one	Antimicrobial, anti-inflammatory, antiproliferative
38.23	C_6_H_6_O_3_	39.9	2-furancarboxaldehyde	Antimicrobial, preservative
43.38	C_4_H_6_O_5_	9.71	Malic acid	Antimicrobial
50.41	C_7_H_6_O	1.41	2,4,6-cycloheptatrien-1-one	Antioxidant

**Table 2:** Binding energies (Kcal) between the FceRI-IgE complex and the bioactive compounds identified in COFE.

Compound	Binding Energy (Kcal)	Van der Waals	Hydrogen Bond	Electrostatic
Loganin	−116.9	−90.5332	−26.3531	0
Cornuside	−141.1	−101.645	−39.4547	0
Naringenin 7-O-β-D-glucoside	−125.7	−96.4237	−29.3185	0

## Supplementary Materials

The following are available online at https://www.mdpi.com/2072-6643/12/11/3317/s1, Supplementary Figure S1: The experimental timeline for β-Hexosaminidase release assay using RBL-2H3 cells (A); NO assay and isolation of iNOS protein (B) and isolation of IkBα and p-p65 protein for Western blot analysis (C) in RAW264.7 cells; flow cytometric analysis for apoptosis assay using HaCaT cells (D). Supplementary Figure S2: Evaluation of the extracts’ 2,2-diphenyl-1-picrylhydrazyl (DPPH) radical scavenging activity (left). After the incubation of the samples and the DPPH solution at 37 °C for 30 min, the absorbance at 517 nm was measured; ascorbic acid was used as a positive control. For the ferric reducing antioxidant power (FRAP) assay (middle), after the incubation of the samples and FRAP reagent at 37 °C for 30 min, the absorbance at 595 nm was measured. For the ABTS assay (right), after mixing the ABTS solution and the samples, the absorbance at 734 nm was measured. The results are expressed as μM Trolox equivalent (TE), Emax, and EC50 (*n* = 3). Letters (a–c) indicate significantly different values (*p* < 0.05), as determined by Duncan’s multiple comparison test. Supplementary Figure S3: Cells were treated with COFE (0–0.3 mg/mL) for 30 min and then treated with LPS (1 µg/mL) for 18 h. The levels of iNOS were determined by Western blotting of the total protein of cell lysates. β-actin was used as a loading control. Values are expressed as the mean ± SD of three independent experiments. Letters (a–d) indicate significantly different values (*p* < 0.05), as determined by Duncan’s multiple comparison test. Reserved DOI:10.5281/zenodo.4065293
