# Supplementary material for: Cornus officinalis Ethanolic Extract with Potential Anti-Allergic, Anti-Inflammatory, and Antioxidant Activities
Source: Nutrients. 2020 Oct 29;12(11):3317. doi: 10.3390/nu12113317 (PMC7692184; doi:10.3390/nu12113317)
Supplement: Supplementary file 1 [file nutrients-12-03317-s001.pdf]

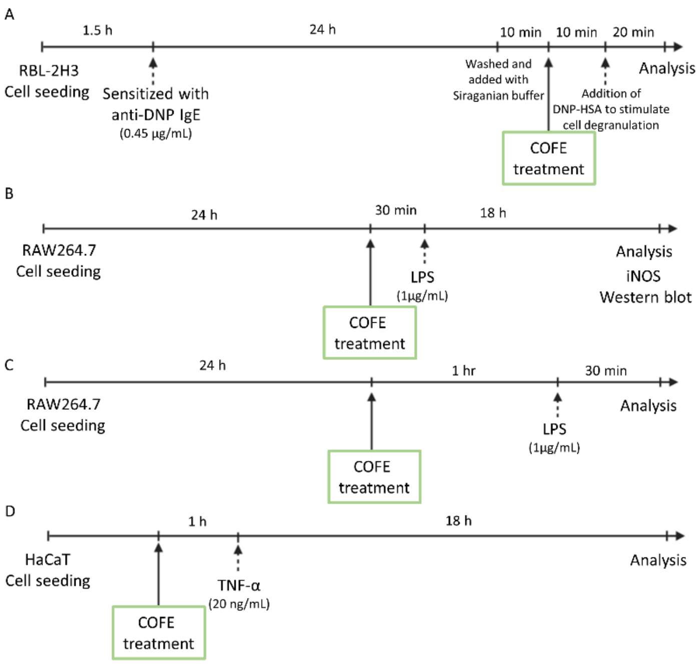

Figure S1. The experimental timeline for β-Hexosaminidase release assay using RBL-2H3 cells (**A**); NO assay and isolation of iNOS protein (**B**) and isolation of IkBα and p-p65 protein for Western blot analysis (**C**) in RAW264.7 cells; flow cytometric analysis for apoptosis assay using HaCaT cells (**D**).

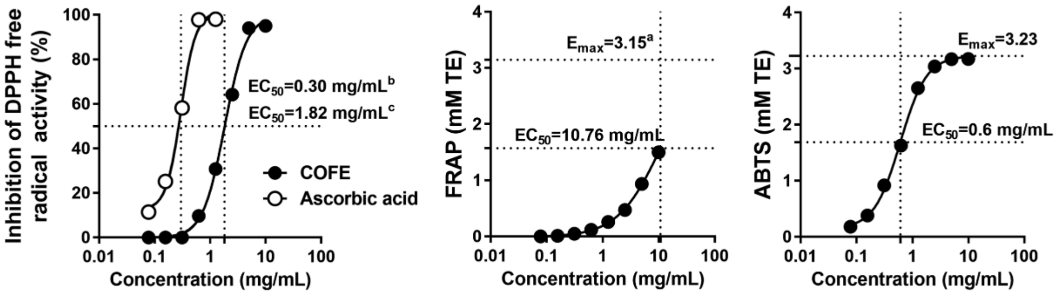

Figure S2. Evaluation of the extracts' 2,2-diphenyl-1-picrylhydrazyl (DPPH) radical scavenging activity (left). After the incubation of the samples and the DPPH solution at 37 °C for 30 min, the absorbance at 517 nm was measured; ascorbic acid was used as a positive control. For the ferric reducing antioxidant power (FRAP) assay (middle), after the incubation of the samples and FRAP reagent at 37 °C for 30 min, the absorbance at 595 nm was measured. For the ABTS assay (right), after mixing the ABTS solution and the samples, the absorbance at 734 nm was measured. The results are expressed as μM Trolox equivalent (TE), Emax, and EC_{50} (n = 3). Letters (a, b, c) indicate significantly different values ($p < 0.05$), as determined by Duncan's multiple comparison test.

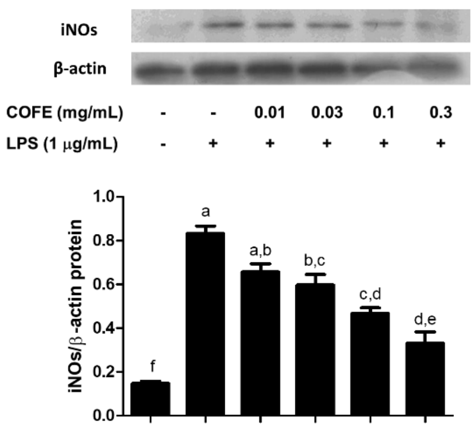

Figure S3. Cells were treated with COFE (0–0.3 mg/mL) for 30 min and then treated with LPS (1 μg/mL) for 18 h. The levels of iNOS were determined by Western blotting of the total protein of cell lysates. β-actin was used as a loading control. Values are expressed as the mean ± SD of three independent experiments. Letters (a, b, c, d) indicate significantly different values ($p < 0.05$), as determined by Duncan's multiple comparison test.
